# A Method of Domain Dictionary Construction for Electric Vehicles Disassembly

**DOI:** 10.3390/e24030363

**Published:** 2022-03-03

**Authors:** Wei Ren, Hengwei Zhang, Ming Chen

**Affiliations:** School of Mechanical Engineering, Shanghai Jiao Tong University, Shanghai 200240, China; perferom@sjtu.edu.cn (W.R.); zhw_SHJD827@sjtu.edu.cn (H.Z.)

**Keywords:** domain dictionary, keyword extraction, terminology, LightGBM, PMI

## Abstract

Currently, there is no domain dictionary in the field of electric vehicles disassembly and other domain dictionary construction algorithms do not accurately extract terminology from disassembly text, because the terminology is complex and variable. Herein, the construction of a domain dictionary for the disassembly of electric vehicles is a research work that has important research significance. Extracting high-quality keywords from text and categorizing them widely uses information mining, which is the basis of named entity recognition, relation extraction, knowledge questions and answers and other disassembly domain information recognition and extraction. In this paper, we propose a supervised learning dictionary construction algorithm based on multi-dimensional features that combines different features of extraction candidate keywords from the text of each scientific study. Keywords recognition is regarded as a binary classification problem using the LightGBM model to filter each keyword, and then expand the domain dictionary based on the pointwise mutual information value between keywords and its category. Here, we make use of Chinese disassembly manuals, patents and papers in order to establish a general corpus about the disassembly information and then use our model to mine the disassembly parts, disassembly tools, disassembly methods, disassembly process, and other categories of disassembly keywords. The experiment evidenced that our algorithms can significantly improve extraction and category performance better than traditional algorithms in the disassembly domain. We also investigated the performance algorithms and attempts to describe them. Our work sets a benchmark for domain dictionary construction in the field of disassembly of electric vehicles that is based on the newly developed dataset using a multi-class terminology classification.

## 1. Introduction

The rapid growth in the market for electric vehicles around the world is essential, and requires the efficient management of obsolete lithium-ion battery packs after completing their service life. According to the work in [1], industrial disassembling is a key enabler of circular economy solutions for obsolete electric vehicle battery systems. However, currently, the battery packs disassembly is primarily accomplished by humans with a fixed robot-assisted battery disassembly workstation. In order to increase the number of electrical vehicles around the world, autonomous robot disassembly of battery packs is imperative.

However, different car manufacturers have adopted different types of battery cell designs and physical configurations, and especially have very diverse scales of disassembly formats and relative sizes, which create a difficulty for battery disassembly automation with robots [2]. Disassembling different battery packs will demand different methods. However, because the disassembled parts, processes, tools, and methods are currently only revealed in text, robots are unable to comprehend the disassembled text’s knowledge on their own. Despite this, robots must be taught to learn from disassembly text on their own.

Nonetheless, robots cannot autonomously understand the knowledge in the disassembled text. So, robot learning from disassembled text is an important research topic that can help the disassembly of electric vehicles, currently handled manually by humans, to allow robotics to take over the task of dismantling. Furthermore, this will improve dismantling efficiency and reduce resources consumption, change the dismantling working environment, reduce worker labor intensity, and increase the annual revenue of the dismantling enterprise. According to the power battery dismantling procedure, that is based on the domain keywords including disassembly parts, disassembly process, disassembly tools, disassembly methods, and other category of disassembly keyword for different disassembly products [3]. Robotics can employ key phrases to decipher the semantic relationships in a disassembly text. However, there is presently no terminology dictionary in the field of disassembly of electric vehicles, thus it is critical and important to create a domain dictionary for data mining from scientific and technological literature in the disassembly domain, which will have enormous academic and commercial value. Furthermore, the goal of creating a domain dictionary automatically has long been a hot topic in NLP research.

Because Chinese words can be made up of multiple characters, and there is no space between them, natural language processing in Chinese is complicated. It’s harder to specify word boundaries properly, especially in the domain of disassembly. Simple and complex phrases are being used in disassembly terminology, with different combinations resulting in different meanings. They are not only rare but also contain a large number of technical terms and proprietary jargon. Due to the lack of a standardized definition of a word from disassembly text, the task of Chinese word segmentation has traditionally begun with the creation of a segmentation standard based on linguistic and task intuitions, followed by the creation of segmenters that output words, and are not suitable for disassembly [4]. For decades, machine learning systems have been used to text feature extraction based on deep learning, but required careful engineering and significant domain expertise to design a feature extractor that transformed the raw data into feature vector. Deep learning learns millions of parameters, features, and feature representations automatically from large data, instead of adopting hand-crafted features, which rely heavily on designers’ past knowledge [5,6]. Recently, rich literature has been produced on machine learning algorithms, and this may be an effective method for text feature extraction. However, the effective information in the disassembled text is relatively sparse.

Rule matches and manual methods are currently one of the most efficient approaches for generating a domain dictionary from text. Then, there’s the massive workload of manually constructing a domain dictionary, and it is difficult to guarantee the coverage of the dictionary. In this paper, we propose a research method that collects keywords from the corpus and then expands the domain dictionary using rules to automatically generate a disassembly domain dictionary.

Currently, there are two types of machine learning algorithms used in keyword extraction research, divided into supervised and unsupervised [7]. Keyword extraction is treated as a binary classification problem in supervised method, and the extraction accuracy of candidate keyword depends on the labeling of the data. However, obtaining the training data set is challenging, and the cost of labeling is high. When faced with a lack of corpus or annotated corpus in the disassembly field, the current extraction method cannot operate effectively [8].

Unsupervised learning methods do not require labeling for keyword extraction, instead relying on TF-IDF and TextRank scores to assess each candidate keyword’s relevance. Unsupervised learning algorithms do not require labeling for keyword extraction, where mainly using TF-IDF and TextRank ranks to measure the importance of each candidate keyword. In recent years, research has focused mainly on adopting more dimensional features to describe the information on keywords as much as possible. However, few studies use graph representations of semantic information for extraction from text. At the same time, unsupervised learning algorithm lacks to consider the impact of additional multi-dimensional features of keyword extraction. In addition, the usually classifiers were naïve Bayes, K-Nearest neighbors, random forest, and others, but they have lower accuracy and performance in the prediction of disassembly domain variables, and require higher memory [9]. In this paper, in order to effectively extract keywords from text and classify keywords into domain dictionary we adopt supervised learning algorithms for keyword extraction from texts, which are based on multi-dimensional features of constructed candidate keywords. To determine whether the candidate keyword is a keyword, we use the LightGBM classification model. The domain dictionary is expanded via pointwise mutual information (PMI).

The remainder of the article is organized as follows: Section 2 is concerned with prior research on the subject; Section 3 provides information on the dataset that we used for our research; Section 4 discusses the study’s methodology, which includes a journey from raw data until terminology categorization; Section 5 is a thorough summary of the results that we achieved, as well as an analysis of the data and explanations for them. Finally, Section 6 concludes the research by summarizing it and suggesting areas for improvement.

The contributions of this paper are as follows:

(1). We propose a method of extracting domain keywords. Firstly, the extraction of disassembly domain keywords is transformed into a machine learning binary classification problem that using disassembly domain keywords and the multidimensional features of constructed candidate keywords. This method is based on the LightGBM classification model, which determines whether the candidate keyword is a keyword.
(2). We expand the domain dictionary based on PMI. The correlation between the keywords in each dictionary is measured by calculating the PMI, with the high correlation between each keyword added to the domain dictionary.

## 2. Related Work

In this paper, we have constructed a domain dictionary algorithm based on multi-dimensional features, and the LightGBM and PMI models are presented. The following is a review of the literature on approaches in the domain dictionary creation algorithm and keyword extraction algorithm.

There are many algorithms for generating domain-specific dictionaries and many scholars who have conducted research on them. In terms of a sentiment dictionary, the POS (part-of-speech) tag is utilized to generate the sentiment dictionary in the field of shopping reviews; the authors in [5] apply POS, occurrence, and frequency to sentiment analysis of user preferences from social media data as well; feature selection and classification are used for a sentiment analysis dataset to recommend movies to other users in [6]; in [10], researchers presented a sentiment analysis-based decision support system by integrating support vector machines with a whale optimization method for autonomously adjusting hyperparameters and conducting feature weighting; the paper in [11] uses topic models, time series analysis, and sentiment analysis to search for rumors in social media texts; in [12], a sentiment analysis of homestay comments dictionary is based on the sentimental PMI algorithm; in [13], a cosine similarity measurement combining word semantic information about TF-IDF method extracts public sentiment keywords from the public opinion on the network.

In other fields, a convolutional neural networks model with the TF-IDF algorithm to extract semantic and location keywords from text for a consumer product defects dictionary has been used [14]. The study in [15] used machine learning, text similarity, and rule-based approaches to mine power field terms and build a professional lexicon in the power dispatching sector.

At present, machine learning algorithms are mainly divided into two categories: Supervised learning and unsupervised learning for keywords extraction [16]. The unsupervised learning method does not require labeling any training data in advance and transforms the task of keyword extraction into a sorting problem.

Unsupervised keyword extraction approaches are classified based on their characteristics into statistics-based methods, graph-based methods, topic-based methods, and language model-based methods, and these methods can be divided into two schools: linguistic school and statistical school. The linguistic school analyzes the topic distribution of articles using linguistic methods. The statistical school focuses on keyword probability features such as TF-IDF and TextRank. Researchers have proposed cross-utilization of the two schools’ knowledge methods of extraction keywords, such as clustering and graphs [16].

### 2.1. Topic-Based Method

Currently, Latent Dirichlet Allocation (LDA) is the most frequently utilized topic modeling approach. It is a probabilistic generative model that characterizes each text as a mixture of topics and each topic as a word distribution [17]. It is a three-level Bayesian model that can extract possible topic models from corpora, provides an efficient method for quantifying research subjects, and is frequently used in text categorization [18]. Many researchers have worked tirelessly to enhance the LDA model in order to achieve the desired topic mining impact. For example, in the field of information security, keywords could well be extracted through using LDA and TextRank models [19]; and LDA models are combined to improve the weight of the essential words [20] adjusting the weights of the keywords’ features from the elements of location and part of speech, and extending the feature generated process of the LDA model to obtain more expressive words.

### 2.2. Statistics-Based Method

The keyword extraction method based on statistical features primarily employs the word weight of terms in the document, word position, and a mixture of word association information to generate a scores for keyword extraction. The features information mainly includes word position, part of speech, word frequency, word length, statistical information, etc. Word positions refer to the distribution information on words with the document, such as title, paragraph beginning, and paragraph end. Word statistical information includes mutual information, mean to value, variance, TF-IDF. The recently popular YAKE model is combines statistical information and context information to extract keywords [16]. Most scholars use a combination of simple textual statistical features to improve the TF-IDF model for extraction keyword. For example: [21] combines discrete coefficients to improve the TF-IDF model, and [22] enhances the TF-IDF model with a fusion word vector to obtain more accurate keywords.

### 2.3. Graph-Based Method

The algorithm TextRank is a graph-based ranking model for keyword extraction from natural language texts [23]. The graph-based ranking model uses the random walk based algorithm, and certain decision rules calculate the keywords weights to achieve extraction from the documents [24], which does not use the external text corpus to enrich the document [25].

At present, the improved TextRank keywords extraction algorithm is based on an improvement model and a fusion model. For improvement of the model, an iterative approach for keyword extraction considers the varied weights of average information entropy, using lexicality into TextRank to enhance the performance of the model, improving weight initialization of the lexical nodes and the transition probability matrix [26]. In addition, improving node initial weights with sentence–sentence, word–sentence, and word—word information characteristics can achieve more excellent extraction results [27].

In terms of the fusion model, in [19] the keyphrase extraction depended on the TextRank model by combining it with the LDA topic model. In [28], an iteration method was based on the transition matrix used the TextRank model for keyword extraction by adding the word vector-based clustering, word embedding model, clustered the nodes, random walk probability, and adjusted the importance of the node position score to improve keyword extraction accuracy.

### 2.4. Language Model-Based Method

In [29], a linguistic features model focuses on a keyword extraction approach by associating parts of speech, N-gram language model, and proper nouns. The study in [30] was based on the N-grams model to discover new words from a large corpus and create a dictionary in the news field. Additionally, ref. [31] improved the accuracy of word segmentation based on the N-gram model with heuristic rules. The paper in [32] improved keyword extraction accuracy for TF-IDF model with location features and N-gram models which adjusted the weight distribution of feature words. To construct a short text feature vector space in emotion information extraction adopts a distributed semantic expression word vector model which was based on Word2Vec algorithm and N-Gram algorithm [33]. The supervised method regards the keywords extraction as a binary classification problem, using classification model to determine whether the candidate keyword is a keyword with the help of multi-dimensional features of words in the text [34]. At present, the keyword extraction research for supervised learning methods is mainly divided into feature extraction and feature classification. Statistical features have been widely adopted in the text including length, frequency, location, as well as linguistic features such as part of speech and syntactic information [16]. Approaches of classification algorithms have different types of text categorization, that mainly include: logistic regression, naïve Bayes, K-nearest neighbors, decision tree, CRF (conditional random fields), random forest, hidden Markov models, maximum entropy models, neural network models and SVM (support vector machine) [7]. The authors in [35] apply a naive Bayes classifier for an automatic news classification problem where TF-IDF algorithm based features are extracted from the news text. To address the problem of poor text sentiment classification accuracy, they adopted Bayesian classification algorithm with feature weighted fusion in sentiment text [36]. In [37], keyword extraction from text based on multi-dimensional features and classifiers in CRF. The work in [38] presents an automatic keyword extraction system with multi-featured supervised learning algorithm and a random forest was used for classifier. In [39], the TextRank algorithm was used to extract keywords from text with multi-features such as TF-IDF, word vector, position, and lexicality, with the help of SVM to train the initial weights of words and as well as to classify keywords and non-keywords.

In summary, the existing works in the literature has achieved some effects, but the multi-feature fusion model for Chinese keyword extraction has not considered the importance of words in the document for the unsupervised method. 

Supervised methods have a higher accuracy than unsupervised methods, but not integrated semantic information into multi-feature fusion model for keyword extraction. Therefore, we propose a supervised keyword extraction algorithm based on multi-dimensional features for the extraction of candidate keywords, with the LightGBM model solving the binary classification problem according to the PMI expansion dictionary.

## 3. Data Description

In this paper, we use the selenium Chrome browser to explore the Chinese dismantling manual, patents and papers in the field of disassembly of electric vehicles, and as the corpus for building the dictionary.

Parts, processes, methods, tools, and other categories of keywords have a specific meaning in the field of disassembly of electric vehicles. The keyword in the literature is clear, concise and precise. We utilize automatic segmentation and labeling with Mandarin Chinese speech corpus and the modern Chinese word segmentation specification for information processing to build a standard corpus in the field of disassembly of electric vehicles. This is based on the stop word list of the Harbin Institute of Technology, which are irrelevantly descriptive Chinese stop words, and of no practical meaning for dismantling in disassembly of electric vehicles.

## 4. Methods

This paper proposes a supervised learning algorithm for Chinese dictionary construction in the field of disassembly of electric vehicles. Candidate keywords extraction based on multi-dimensional features, and LightGBM to classify Candidate keywords, then the dictionary will automatically extend with PMI.

**Figure 1:**
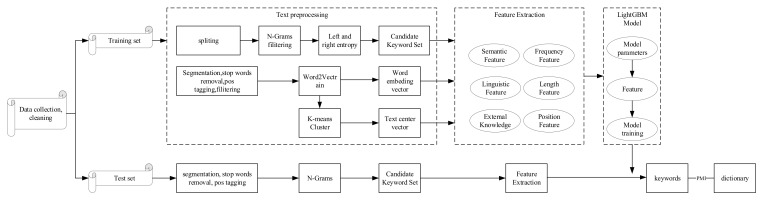
Flow chart of model.

Our method is based on multi-dimensional features such as position, linguistic, length, term-frequency, external knowledge, semantic features, length, and other features to extract candidate keywords from the corpus in the disassembly field, using the LightGBM model to determine whether the candidate keyword is a keyword. Computing keywords are associated with PMI and automatically expand the domain dictionary. Figure 1 shows the flow of the entire algorithm.

### 4.1. Text Preprocessing

In order to obtain a set of candidate keywords that it is necessary to arrange the input corpus by degree of impact according to appear in the first, middle and last paragraphs of a keyword and in the titles [21,40]. The weight for degree of impact for corpus as (1)

(1) $${\mathrm{pos}}_{\mathrm{ij}}\left\{\begin{array}{cc} 1 & \mathrm{in}\;\mathrm{the}\;\mathrm{titles}\\ 0.5 & \mathrm{the}\;\mathrm{begin}\;\mathrm{and}\;\mathrm{end}\;\mathrm{of}\;\mathrm{paragraphs}\\ 0.2 & \mathrm{other}\;\mathrm{position} \end{array}\right.$$

First, we follow the first step of the Chinese natural language processing process to segmentation corpus and remove stop words that using the Chinese word segmentation tool of Jieba, and place the disassembly vocabulary in the Jieba directory. In most cases, the length of disassembly words does not exceed 6 and the n is proposed as 6 to cover all possible keywords as comprehensively as possible [41]. The paper in [42] filters candidate keywords from n-grams with part-of-speech and obtains a candidate key word set, then discovers new words with mutual information and left-right adjacency entropy in the field of disassembly, and then filters unreasonable new words according to the score. The calculation formula of the mutual information is Formula (2); the calculation formula of the left adjacency entropy is Formula (3); the calculation formula of the right adjacency entropy is Formula (4).

(2) $$\mathrm{MI}\left(x,y\right)=\log\frac{p\left(\mathrm{xy}\right)}{p\left(x\right)p\left(y\right)}$$

Here, p(x) and p(y) indicates the probability of x and y appearing in the separately corpus; p(xy) indicates the probability that x and y appear together in the corpus, if MI(x,y) > 0, it means that x and y are closely related that the larger of the value and the more likely which become a new word. If MI(x,y) = 0, means that x and y are distributed independently of each other. If MI(x,y) < 0 it means that x and y are not related.

Left Entropy

(3) $$\mathrm{HL}\left(x\right)=-\underset{a}{\sum }p\left(a|x\right)\log p\left(a|x\right)$$

Right Entropy

(4) $$\mathrm{HR}\left(x\right)=-\underset{b}{\sum }p\left(b|x\right)\log p\left(b|x\right)$$

Here, p(a|x) indicates the probability that a is the left adjacent character of the candidate word x; p(b|x) indicates the probability that b is the right adjacent character of the candidate word x.

### 4.2. Feature Extraction

Feature extraction is based on feature engineering that correctly distinguishes between keywords and non-keywords from the candidate keywords sets. Keyword extraction is based on multi-dimensional features, which combines position feature, linguistic features, length feature, term-frequency feature, external knowledge-based features and semantic features to extraction the candidate keywords from the corpus.

#### 4.2.1. Position Feature

Keywords often appear in important positions, such as the beginning of a paragraph and the end of a paragraph. In this paper, keywords appear in the titles, beginning, middle and end of a paragraph as position features. Position described the importance of keywords (see in Table 1).

#### 4.2.2. Linguistic Feature

Because the keywords have a specific part of speech in the field of disassembly, we can identify lexical features of the terminology under part of speech or proper nouns [43].

#### 4.2.3. Length Feature

In this paper, the length feature refers to the length of the candidate keyword itself and the sentence in which it is located, and refers to the number of words contained in the candidate keywords [44]. Because the length of the keyword is usually less than or equal to the length of 6, it has a good distinction. 

Average sentence length refers to the average number of words of all sentences containing candidate keywords. The maximum length and the minimum length of the keywords where the number of words of all sentences containing candidate keywords [43,45].

(5) $${\mathrm{wl}}_{i}=\frac{\mathrm{length}\left(i\right)-\mu }{\sigma }$$

$\mathrm{length}\left(i\right)$ indicates the length of the keyword, μ indicates the average of the length of all keywords, σ represents the variance of the length of all keywords.

(6) $${\mathrm{sl}}_{s}=\frac{\mathrm{length}\left(i\right)-\mathrm{shortest}\left(i\right)}{\mathrm{lengest}\left(i\right)-\mathrm{shortest}\left(i\right)}$$

$\mathrm{length}\left(i\right)\;$ indicates the number of words contained in the sentence *i*, $\mathrm{shortest}\left(i\right)$ represents the number of words contained in the shortest sentence in the text *i*, $\mathrm{lengest}\left(i\right)$ represents the number of words in the longest sentence in the text *i*.

#### 4.2.4. Term-Frequency Feature

The term frequency refers to the frequency of words or phrases appearing in a given document. It is generally believed that the more frequently a term appears and the more significant it is. However, there are some exceptions where the frequency of some words is high but not important and there are some sparse data but also very important. In this paper, head word frequency, term frequency, inverse document frequency, TF-IDF and title word frequency to measure the importance of candidate keywords [44] (Table 2).

#### 4.2.5. External Knowledge-Based Feature

This paper employs external knowledge-based features to measure the importance of candidate keywords, and we believe that the candidate keywords which can be searched in the domain dictionary are more essential. If the candidate keyword exists in the domain dictionary as a whole that all occurrence is 1, the partial occurrence is 0.5, and the non-appearance is 0.

#### 4.2.6. Semantic Feature

In this paper, semantic feature is one key point of feature to measure the importance of candidate keywords. Word2vec model converts words to their corresponding vectors into n-dimensional space to representation of any particular word. Word2Vec can provide an efficient implementation of architectural CBOW (continuous bag of words) and Skip-Gram to calculate vector representations of words. For a small amount of the training data, that CBOW has slightly better accuracy for Skip-Gram, so we combined CBOW model to predict the word in training sets. Along with their distance similarity index as semantic feature. The smaller the distance, the greater the similarity and the closer the candidate keyword to the semantic relation [46]. The cosine similarity formula is as follows:

(7) $$\mathrm{Similarity}=\cos\theta =\frac{\;\bar{a}\times \;\bar{b}}{\;∥\bar{a}∥\;∥\bar{b}∥}$$

where: $\;\bar{a}\times \;\bar{b}$ vector dot product from a and b. $\sum_{k=1}^{n}a_{k}b_{k}$, $∥\bar{a}∥$: long vector a. $\sum_{k=1}^{n}a_{k}{}^{2}$,$\;∥\bar{b}∥$: long vector a.$\;\sum_{k=1}^{n}b_{k}{}^{2}$.

Cluster-Based keyword extraction uses the K-Means clustering algorithm to achieve the k topic words as initial clustering centers that calculate the distance between each candidate keyword and each clustering center based on multi-dimensional features. In order to get more reasonable clustering centers, in this paper, we choose more weight top k words as the initial cluster center. The weights of keyword formula as follows:

(8) $$w_{i,j}=\alpha \times {\mathrm{tfidf}}_{i,j}+\beta \times {\mathrm{ttf}}_{i,j}+\gamma \times {\mathrm{span}}_{i,j}$$

$w_{i,j}$ represents the weight of the keyword i in the disassembled text j, ${\mathrm{tfidf}}_{i,j}$ represents the term frequency and inverse document frequency, ${\mathrm{ttf}}_{i,j}$ represents the term frequency in the title,${\;\mathrm{span}}_{i,j}$ indicates the length between the first and last occurrence of word in a text. The parameters α, β, and γ indicate that the weight coefficients are 0.3, 0.5, and 0.2 respectively.

(9) $${\mathrm{tfidf}}_{i,j}\;=\frac{n_{i,j}}{\left|j\right|}\times \log_{2}\frac{\left|D\right|}{{\mathrm{df}}_{i}}$$

(10) $${\mathrm{ttf}}_{i,j}=\frac{n_{i,j}}{\left|j\right|}$$

(11) $${\mathrm{span}}_{i,j}=\frac{{\mathrm{pos}}_{-1}\left(i,j\right)}{\left|j\right|}-\frac{\mathrm{pos}\left(i,j\right)}{\left|j\right|}$$

$n_{i,j}$ represents the number of times the keyword i appears in the text j. $\left|j\right|$ indicates the number of keywords in the text. $\left|D\right|$ indicates the number of texts in the corpus. ${\mathrm{df}}_{i}$ represents the number of texts containing the keyword i  in the corpus. ${\mathrm{pos}}_{-1}\left(i,j\right)\;\mathrm{and}\;\mathrm{pos}\left(i,j\right)$ respectively represent keyword i  in the position of the last occurrence and first occurrence in the text j. 

Then each keyword and each cluster center according to Euclidean distance to clusters.

(12) $$\mathrm{dis}\left(v_{i},c_{j}\right)=\sqrt{{\left(v_{i,1}-c_{j,1}\right)}^{2}+\cdots +{\left(v_{i,d}-c_{j,d}\right)}^{2}}=\sqrt{\sum_{t=1}^{d}{\left(v_{i,t}-c_{j,t}\right)}^{2}}$$

$v_{i}$ represents the long vector i, 1 ≤i≤m. $c_{j\;}$ represents the cluster center of j, 1≤i≤k. $v_{i,t}$ represents the dimensional of attribute of the *i* keyword word vector.

### 4.3. Classification

#### 4.3.1. LightGBM

LightGBM is a weak learner as a regression tree base on gradient boosted decision trees. The gradient boosting means sequentially combining weak learners in a way that each new learner fits the residuals from the previous step. Thus, each new learner improves the overall model. The final model aggregates the results from each step, and a strong learner is achieved. We train LightGBM algorithm and split a data sample using k-fold cross-validation to keyword extraction from validation sets in the field of disassemble.

For position features, semantic feature, linguistic feature, external knowledge-based features, statistical features, term frequency, length feature, and other multi-dimensional features are finally connected into multi-dimensional feature vectors, and the feature vectors are used to complete the training of the LightGBM classifier. At this time, while making a decision tree, the position of each leaf node is 0, otherwise, it is set to 1.

(13) $$x_{i}^{\prime }=g{\left(x_{i},\theta \right)}_{\mathrm{num}\_\mathrm{tree}\times \mathrm{num}\_\mathrm{leaves}}$$

$x_{i}^{\prime }$ represents the high-dimensional combination 0–1 feature vector of the training sample  i; $x_{i}$ represents the feature vector of the training sample i; $g\left(\cdot \right)$ represents the leaf node of the LightGBM classifier when the sample i belongs to leaf nodes is 1, otherwise 0; num_tree represents the number of decision trees in the LightGBM model; num_leaves represents the number of leaf nodes on each decision tree.

#### 4.3.2. PMI

PMI is a measure of association used in information theory and statistics. It can be used as a measure to determine whether the keyword on the category based on the assumption that keywords and category’s have similar [47,48]. The PMI calculation depends on words $w_{1}\;$ and $w_{2}$ is as follows.

(14) $$\mathrm{PMI}\left(w_{1},w_{2}\right)=\log_{2}\frac{P\left(w_{1},w_{2}\right)}{P\left(w_{1}\right)P\left(w_{2}\right)}$$

## 5. Design and Analysis of Experiments

### 5.1. Datasets and Evaluation Indicators

This paper selects construction dictionary in the field of disassembly of electric vehicles to verify the results and performance of the algorithm. This paper collects the China’s latest academic patents and papers and disassembly manual in the field of disassembly the electric vehicle with the selenium Chrome browser by ‘disassembly electric vehicle’ keywords. In construction dictionary study, the search yielded a total of 1230 academic articles and 5 Disassembly Manual, the corpus contains text, title, abstract, and keyword. Splitting corpus into training set and test set according to 4:1, in order to evaluate the performance of our model that combines precision rate P, recall rate R and $F_{1}$ value for the classification results. The calculation formulas for the three evaluation indicators are as follows:

(15) $$P=\frac{A}{A+B}$$

(16) $$R=\frac{A}{A+C}$$

(17) $$F_{1}=\frac{2\mathrm{PR}}{P+R}$$

where A indicates that the number of keywords extracted is correctly identified, B indicates that the number of keywords extracted, C indicates that the number of label keywords. The experiment in this paper is carried out under the Ubuntu 20.04 LTS system, The CPU is Inter Core i5-3230M 2.6 GHz, the memory size is 16 G, the experimental programming language is Python3.6, the development tool is Visual Studio Code, and the deep learning framework used is Tensorflow1.2.0.

### 5.2. Experimental Setup

In this paper, the corpus data for disassembly of electric vehicle processing with UTF-8 encoding format. Add the stop word list of Harbin Institute of Technology, irrelevant descriptive and no practical meaning in the field of dismantling to Jieba tokenizer as Chinese stop words in disassembly of electric vehicle. Calling the Jieba in Python for word segmentation remove stop words, irrelevant descriptive word, and no practical meaning from the corpus. In our experiments, the CBOW parameter settings of the model are shown in Table 3.

Due to the variable number of topics contained in corpus data, therefore, the number of clusters k cannot be determined. In order to select the appropriate number of clusters that we use the different number of keywords k in the range of 3 to 8 to verify the performance of precision, see in Table 4. 

Light GBM is sensitive to overfitting and can easily overfit for handling the small size of data and takes lower memory to run. This paper extracts multi-specific features in different aspects, the LightGBM parameter settings of the model are shown in Table 5.

### 5.3. Comparison of Results for Different Feature

**Figure 2:**
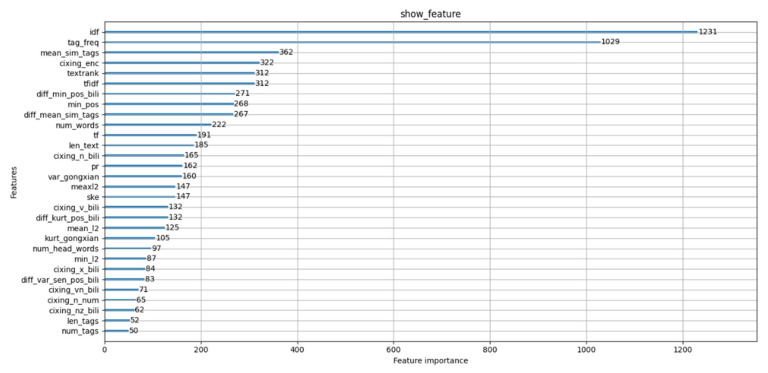
Comparison of results for different features.

In this paper, we proposed based on Information Gain to measures the importance of multi-dimensional features. Figure 2 shows that the influence of multiple features of the extraction results where IDF features, title frequency, semantic features, part of speech features have more influence than other features.

For verification of the performance of extracting the number of different keywords, we select the number of keywords in the range of 3 to 8, and a comparative experiment was design for extracting a different number of different keywords. Table 4 shows the multi extraction results with the number of keywords, and as the number of keywords increases, the recall continues to decrease and the precision continues to increase for the F1 score, which can better reflect the performance of the algorithm and that increases first and then decreases when the number of keywords is 5 and the performs is best. By comparison we find that the word count for professional vocabulary in the field of disassembly is around 5.

In order to verify the performance of our algorithm, we conduct a comparative experiment, as shown in Table 6, confirming that our model is significantly better than classic keyword extraction algorithms, such as TFIDF, TextRank, BERT and YAKE. The experiment proves that keyword extraction performs better for precision and F1 score, with the precision value increased to 0.95. Because of the vast amount of proprietary jargon in the disassembly document, as well as the lack of emotion in the depiction of scientific and technical literature. In addition, there is no obvious semantic relationship in the scientific and technical literature. Although the BERT model is more accurate than previous models, it takes a long time to train, and our model can meet the real-time requirements.

For the performance of the classification algorithm for LightGBM we are using SVM, K neighbors, and random forest as comparisons, as seen in Table 7. From the Table 7, it can be observed that the classification algorithm LightGBM is significantly better than classic keyword extraction algorithms. Combining the features of the LightGBM model and disassembly text that LightGBM is more suitable for classification on the domain of disassembly of electric vehicle for construction dictionary.

### 5.4. The Result of Dictionary Construction

To every keyword we get from the classification algorithm, we measure the terms polarities by PMI value. For every category on Dictionary that we adopt PMI value is greater than zero word and word is relation, the PMI value is equal to zero word and word is mutually independent for each other, the PMI value less than zero to be mutually exclusive. After data analysis (see in Table 8), we found the largest number of parts in the document was extracted by PMI and there is relatively little terminology for methods. The Extraction accuracy of PMI conforms to the distribution of keywords in the text, which means that there are more keywords will be found by our algorithm model if try it in more text.

### 5.5. The Result of Extraction for Model

To compare the overall performance of our models for building dictionaries, we take different model, such as TFIDF + SVM + PMI, TextRank + random forest + PMI, BERT + LightGBM + PMI, Word2Vec + LightGBM + PMI, etc. The data is presented in Table 9, our model has significant advantages in dictionary construction for disassembly of electric vehicle. We received more accurate results as we increased the number of features supplied to the classifiers. Furthermore, semantic information exceeds other features in terms of extraction performance. The argument is similar to that of a previous observation: as NN-based embeddings, Word2Vec and BERT can provide richer semantics even with a smaller dataset. Word semantics were better captured in these word embeddings with richer vocabularies and a larger corpus. At the same time, different features and classification algorithms present different extraction results. The combination of LightGBM and PMI beats other combinations.

## 6. Conclusions

In this paper, we respond to the challenge of the lack of a domain dictionary in the field of electric vehicle disassembly and traditional domain dictionary construction algorithms that do not effectively extract terminology from disassembly text, because the terminology is complex and variable. We proposed a method for automatic dictionary construction in the field of electric vehicle disassembly, with each candidate keywords extraction based on multi-dimensional features, and then proposed LightGBM to quantify the relevance of candidate words, with automatic dictionary extensions using PMI that combines position feature, linguistic features, length feature, term-frequency feature, external knowledge-based features, semantic features, and other multi-dimensional features extraction for the keywords from the disassembly corpus. Based on the multidimensional features, we describe word information more comprehensively and explain word importance more completely. Additionally, the LightGBM can identify keywords in an accurate, efficient, and consistent manner. Finally, we designed a PMI model that identified the various types of keywords. The experimental results show that our model can significantly improve extraction and classification performance. Compared with other models, our model is more suitable for identifying diverse features of keywords, classification, and expansion, and its accuracy is obviously higher than the other models. For the next step, we will focus on higher expected performances using BERT-BiLSTM-CRF, leaving this exploration for future work.

## Figures and Tables

**Table 1:** Position Feature.

First occurrence of word in a text	$\mathrm{FP}\left(p,d\right)=\frac{\mathrm{pos}\left(p,d\right)}{\left|d\right|}$	$\mathrm{FP}\left(p,d\right)$ is the relative position of the first occurrence d
Last occurrence of word in a text	$\mathrm{LP}\left(p,d\right)=\frac{{\mathrm{pos}}_{-1}^{\left(p,d\right)}}{\left|d\right|}$	$\mathrm{LP}\left(p,d\right)$ is the relative position of the last occurrence

**Table 2:** Feature type and description.

Type	Equation	Describe
TF	${\mathrm{tf}}_{i,j}=\frac{n_{i,j}}{\left|j\right|}$	$n_{i,j}$ represents the number of times the word i appears in the document j, and $\left|j\right|$ represents the number contained in the document j
IDF	${\mathrm{idf}}_{i}$ = $\log_{2}\frac{\left|D\right|}{{\mathrm{df}}_{i}}$	$\left|D\right|$ represents the number of texts contained in corpus D, and ${\mathrm{df}}_{i}$ represents the number of texts in the corpus containing word i
TF-IDF	${\mathrm{tfidf}}_{i,j}$ = ${\mathrm{tf}}_{i,j}\times {\mathrm{idf}}_{i}$	————
TTF	${\mathrm{ttf}}_{i,j}$ = $\frac{n_{i,j\;\bar{t}}}{\left|j\;\bar{t}\right|}$	$n_{i,j\;\bar{t}}$ represents the number of times the word i appears in the title of the text j, and $\left|j\;\bar{t}\right|$ represents the number of words contained in the title

**Table 3:** The parameter settings of the CBOW model.

Parameter	Size	Window	Min_Count	CBOW_Mean	Sample
Value	100	10	5	1	0.0001

**Table 4:** Comparison of results for different k.

k	Precision	Recall	F1
3	0.36	0.64	0.47
4	0.41	0.65	0.48
5	0.45	0.62	0.53
6	0.47	0.58	0.52
7	0.54	0.49	0.50
8	0.55	0.46	0.49

**Table 5:** Parameters required for LightGBM.

Parameters	Boosting_Type	Objective	Metric	Num_Leaves
Value	Gbdt	Binary	Binary_Logloss, Auc	5
Parameters	Max_Depth	Min_Data_In_Leaf	Learning_Rate	Feature_Fraction
Value	6	450	0.1	0.9
Parameters	Bagging_Fraction	Bagging_Freq	Reg_Alpha	Reg_Lambda
Value	0.95	5	1	0.001
Parameters	Min_Gain_To_Split	Verbose	Is_Unbalance	— —
Value	0.2	5	TRUE	— —

**Table 6:** Comparison of results for different keyword extraction algorithms.

Num	Algorithm	Precision	Recall	F1
1	TFIDF	0.66	0.55	0.65
2	TextRank	0.35	0.42	0.47
3	YAKE	0.41	0.49	0.43
4	TFIDF- TextRank	0.75	0.65	0.64
5	BERT	0.83	0.71	0.69
6	CBOW	0.63	0.64	0.58
7	Skipgram	0.64	0.66	0.61
8	Multi-Dimensional Features	0.95	0.61	0.78

**Table 7:** The result for difference classification algorithm.

Classification Algorithm	Precision	Recall	F1
SVM	93.65	92.60	92.59
K neighbors	93.65	91.63	92.66
Random forest	94.96	92.94	93.95
LightGBM	99.69	99.54	99.42

**Table 8:** The result for difference PMI value.

Terms	PMI	Size
Parts	0	410
Processes	0	118
Methods	0	94
Tools	0	195
Other	0	48

**Table 9:** The result for difference model algorithm.

Extraction Algorithm	Precision	Recall	F1
TFIDF + SVM + PMI	90.12	89.63	91.65
TFIDF + random forest + PMI	90.55	90.15	90.57
TFIDF + LightGBM + PMI	90.88	90.79	90.68
TextRank + SVM + PMI	92.23	92.45	91.57
TextRank + random forest + PMI	92.56	92.89	91.67
TextRank + LightGBM + PMI	93.01	92.50	92.60
BERT + SVM + PMI	94.05	93.44	92.87
BERT + random forest + PMI	94.36	92.14	92.73
BERT + LightGBM + PMI	94.89	93.58	92.91
Word2Vec + SVM + PMI	93.16	92.57	92.12
Word2Vec + random forest + PMI	93.46	93.01	91.25
Word2Vec + LightGBM + PMI	93.89	92.45	91.45
Our Model	98.02	95.55	95.83

## Data Availability

The entire dataset come from latest academic patents and papers and disassembly manual in the field of disassembly the electric vehicle.
